# Surface similarity-based molecular query-retrieval

**DOI:** 10.1186/1471-2121-8-S1-S6

**Published:** 2007-07-10

**Authors:** Rahul Singh

**Affiliations:** 1 Department of Computer Science, San Francisco State University, San Francisco, CA 94132, USA

## Abstract

**Background:**

Discerning the similarity between molecules is a challenging problem in drug discovery as well as in molecular biology. The importance of this problem is due to the fact that the biochemical characteristics of a molecule are closely related to its structure. Therefore molecular similarity is a key notion in investigations targeting exploration of molecular structural space, query-retrieval in molecular databases, and structure-activity modelling. Determining molecular similarity is related to the choice of molecular representation. Currently, representations with high descriptive power and physical relevance like 3D surface-based descriptors are available. Information from such representations is both surface-based and volumetric. However, most techniques for determining molecular similarity tend to focus on idealized 2D graph-based descriptors due to the complexity that accompanies reasoning with more elaborate representations.

**Results:**

This paper addresses the problem of determining similarity when molecules are described using complex surface-based representations. It proposes an intrinsic, spherical representation that systematically maps points on a molecular surface to points on a standard coordinate system (a sphere). Molecular surface properties such as shape, field strengths, and effects due to field super-positioningcan then be captured as distributions on the surface of the sphere. Surface-based molecular similarity is subsequently determined by computing the similarity of the surface-property distributions using a novel formulation of histogram-intersection. The similarity formulation is not only sensitive to the 3D distribution of the surface properties, but is also highly efficient to compute.

**Conclusion:**

The proposed method obviates the computationally expensive step of molecular pose-optimisation, can incorporate conformational variations, and facilitates highly efficient determination of similarity by directly comparing molecular surfaces and surface-based properties. Retrieval performance, applications in structure-activity modeling of complex biological properties, and comparisons with existing research and commercial methods demonstrate the validity and effectiveness of the approach.

## Background

Across all biological and pharmaceutical investigations, the discovery (or development) of molecules with desired biological activity is an important goal. Efforts to attain this goal are strongly driven by the notion of molecular similarity because in general similar molecules tend to behave similarly [1,2]. Effective determination of molecular similarity requires accounting for both structural and physicochemical characteristics of molecules [3]. It is therefore closely related to the notions of molecular representation and molecular descriptors. We begin this section with a review of techniques for molecular representation and molecular descriptors. Next, we outline and discuss different formulations of the molecular query-retrieval problem. This is followed by a review of the prior research in this area. The last subsection introduces the problems associated with determining molecular similarity using complex 3D surface-based descriptors

### Introduction to molecular representations and descriptors

**Figure 1:**
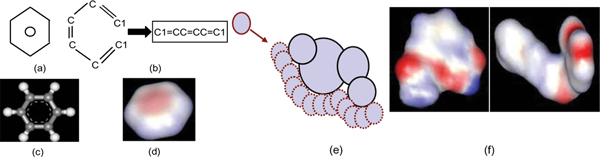
**Molecular Representations**. Different molecular representations shown with the Benzene molecule as an example: (a): chemical (graphical) representation, (b) 2D graph and graph-traversal based string representations, (c) 3D graph-based representation, (d) surface-based representation. The molecular surface is obtained by rolling a probe-atom over a molecule as shown in (e). The complexity of surface-based representations can be discerned from (f) where the molecule Asprin in shown on the left and the molecule Capceisin on the right.

In their simplest form, molecules can be represented using chemical formulae. However, different structures may yield the same formula even though they possess dissimilar physical or biochemical properties (e.g. in the case of isomers). Therefore, commonly employed representation frameworks tend to emphasize a more explicit characterization of the molecular structure and include (see Figure 1): (1) one-dimensional string-based descriptors, such as *SMILES *obtained by ordered traversal of the molecular graph, (2) vector-space representation of (typically structural) attributes of a molecule called *structure keys *that encode presence/absence of predefined sub-structural motifs in the molecule in a binary string, (3) two-dimensional and three-dimensional graphscharacterizing molecular connectivity and inter-atomic distances, and (4) three-dimensional surface based representations, such as the Connolly surface. The Connolly surface is obtained by rolling a probe-atom over the molecule and is defined as the set of points where the surface of the probe atom touches the *van der Walls *surfaces of the atoms in the molecule. It may be noted that the complexity of representations is directly correlated with their fidelity in describing biochemical characteristics of molecules. For example, simple characteristics of molecules such as their atomic weight or connectivity can be derived from SMILE strings. However, more complex biochemical properties like molecule-molecule interactions or permeation through membranes are more accurately modelled using surface-based representations [4-8].

Molecular descriptors are computationally determinable characteristics of a molecule that describe specific molecular properties. Examples include physical-chemical descriptors such as the number of rotatable bonds, polar surface area, electronegativity, descriptors of molecular connectivity such as the Wiener number [9], the Randic index [10], structure keys and molecular fingerprints, eigenvalue-based descriptors [11], molecular moment-based descriptors such as CoMMA [12], and surface and field-based descriptors [4]. Other descriptors include donor-acceptor atoms [13] and those based on the molecular wave/density functions [14]. Modern approaches to correlating molecular structure with biological activity emphasize the use of molecular *fields *(see for instance [15] and references therein); given any molecular property *P *that can be calculated at an arbitrary point around a molecule, a field can be created by integrating *P* with respect to volume. Field-based descriptors typically are superposition-based, in that their value at any particular point, takes into account the influence of multiple atoms of the molecule at that point. It is also straightforward to define field-based descriptors at the molecular surface, thereby incorporating both physicochemical and physically-relevant structural attributes in a single framework.

### Formulations for molecular query-retrieval and analysis of prior research

The problem of molecular query-retrieval can be approached from two primary and interrelated perspectives:

#### • Query formulation

Two main forms of formulating the query can be distinguished: (1) *Sub-structure-based query*, where the query-structure is constrained to be a proper subset of each of the retrieved structures. (2) *Whole-molecule query*, where molecules are retrieved in terms of their overall similarity, as defined using appropriate similarity functions, to the query molecule. It is important to note that sub-structure searching requires the user to have a clear picture of the structures which are to be retrieved prior to issuing the query [16]. Typically such detailed knowledge is available only when the mechanism of action of the molecule is established in terms of its activity as determined by specific structural fragments. In contrast, "whole molecule" similarity is suitable for exploring structural space [16], generating hypotheses, or querying chemical databases when detailed structure-activity information, at the level necessary for sub-structure querying is unavailable.

#### • Molecular representation

Molecular representations have varying capabilities in terms of modelling biochemical characteristics of molecules. As noted earlier, surface-based representations/descriptors are more faithful to the actual physics of molecules than molecular graphs-based approaches [4,5,7,8,17]. At the same time, graph-based representations, owing to their graphical similarity to chemical notations, tend to be highly intuitive and are also computationally easier to characterize.

Early attempts at determining molecular similarity, like [9,10], used variations on the sum of inter-atomic distances. Later approaches have looked at schemes for atom re-labelling to minimize a difference-distance matrix or decomposing the molecular distance and connectivity graphs into sub-graphs which are numerically characterized and compared [18]. Other efforts have tried to characterize similarity of molecular graphs, using edit distances, frequent sub-graphs [19], or maximal common sub-graphs [20]. These techniques either focus on building structure-property models (and are inapplicable to query-retrieval formulations) or do not efficaciously scale up with repository size. With our research goals in mind, at the state-of-the-art, two classes of techniques merit discussion:

*1. Matching techniques using fixed-size representation vectors*: have been amongst the most efficient and are employed almost in all small molecule repositories of significant size. In this approach a molecule is represented using a fixed size vector. Each element of the vector encodes for the presence (or frequency) of a predefined attribute, for example, specific structural motifs [21] or the unique labelled paths obtained during a traversal [22]. The vectors are then compared using well established dissimilarity measures such as the Hamming, Euclidean, and Tanimoto measures.

*2. Matching techniques using 3D molecular graphs: *depend on super-positioning the 3D graphs of the molecules being compared. Significant research in this context has been done of aligning structures of large (protein) molecules leading to techniques such as DALI [23], SSM [24], SSAP [25], STRUCTAL [26], CE [27], LOCK [28], and LSQMAN [29]. Other efforts include the application of *geometric hashing *and its variations [8]. In [4,7,17], molecular similarity is defined using surface and field characteristics: First, the field-effects around a molecule are estimated. Then, the orientation of the query/model molecule (a 3D graph), is varied to minimize an RMS error between the field values.

The use of fixed-size representation vectors has lead to practical solutions for querying large molecular repositories. However, such approaches have several severe drawbacks: (1) They are limited to 2D information and incapable of being used for complex bio-chemically relevant representations/descriptors. (2) They are incapable of representing *bioisosteres *(structurally different molecules exhibiting the same biological effect). (3) Such representations are predefined rather than being data-driven. Therefore, they are incapable of capturing specificities of molecules which were not preconceived. On the other hand current approaches to 3D matching simply don't scale with respect to repository size and time constraints typical to modern query-retrieval formulations. Moreover, such approaches, even when they seek to compare surface-based descriptors, do so indirectly. That is, the 3D graphs are superimposed and only then are the respective surfaces compared. Such an approach can miss molecules which have similar surface/field-based properties, but whose 3D structures do not necessarily superimpose well.

### Problem characteristics and challenges

The problem of determining the similarity of molecules when they are represented using complex 3D surface-based descriptors presents some unique challenges which include:

1. *Definition of a standard coordinate system for surface-based molecular representations*: To compare molecules using their surface-based descriptions, it is necessary to have a way of representing the shape of their surfaces. The complexity lies in defining an intrinsic (view independent) coordinate system over the curved molecular surface that maps a point on the curved surface to a point on a standard coordinate system. Additionally, such a mapping should be one-to-one between points on the molecular surface and the standard coordinate system.

2. *Multi-modal nature of molecular properties*: Molecular properties like geometry and donor/acceptor fields have entirely different characteristics. For example, while the geometric representation of a molecule is unique, donor/acceptor fields are superposition-based. Representation frameworks need to account for such issues.

3. *Query efficiency*: It is typical to conduct molecular similarity queries over large sets ranging from thousands to millions of molecules. The latter order of magnitude is especially common in pharmaceutical settings. It is therefore imperative for similarity determination approaches to be computationally efficient.

## Results

Three different types of experiments were conducted to study the efficacy of the proposed method: (1) Investigation of the method's accuracy in query-retrieval settings, (2) Evaluation of its performance (speed), and (3) Validation through applications in structure-activity modelling problems. Each experiment incorporated two stages: The first stage involved a direct application of the method on a data set with subsequent analysis of the results. In the second stage, a comparative study was performed by applying a state-of-the-art research or commercial technique on the same data set. Subsequently the results were analysed to evaluate the proposed approach.

### Accuracy in query-retrieval settings

The method was tested in a query-retrieval setting on a subset of 5000 molecules randomly selected from the MDDR collection [21]. The MDDR collection consists of molecules that are either marketed drugs or have reached advanced stages in a drug discovery process. Each of the 5000 molecules was successively used as a query against the rest of the molecules. The query and model molecules were each represented by 20 conformers, i.e. 400 distinct molecular conformers were used per similarity computation. Since the proposed method does not require super-positioning of the underlying structures, to distinguish its performance from approaches that do so, a variation of the experiment was performed where the query was represented by 20 novel (distinct from the model) conformers. It may be noted, that for some molecules, 20 novel energetically stable conformers could not be obtained. In such cases, as many novel conformers as could be derived for each specific structure were used. In the second stage of this experiment, for purposes of comparison, the query-retrieval experiments were performed using ISIS [21], a widely used commercial 2D chemical database. ISIS uses structure-keys in conjunction with indexing for answering queries. However, molecular similarity using ISIS is strictly 2D-substructure-based and can not incorporate issues like conformations. The consolidated results from these two stages are presented in Table 1. The first row of the table shows results obtained with ISIS. The second row presents the results obtained with 20 conformers for each of the query and model molecules. The final row shows the accuracy of the retrieval process when distinct conformers (between the query and the model) were employed. Here, the asterisk denotes the aforementioned fact that for some molecules 20 distinct stable conformers were not obtained. In this setting, of the 5000 molecules, 4910 were correctly identified. An analysis of the results obtained in this step indicates that the accuracy of the proposed approach during query-retrieval is comparable to that of ISIS, even though the proposed method addresses the query-retrieval problem in a setting that involves molecular conformations, surface-properties, and superposition-based effects and is therefore much more complex than the 2D structural motif-based search used in ISIS.

**Table 1:** Summary of results from the query-retrieval experiment.

**Method**	**Data Size**	**Number of Conformations**	**Accuracy**
ISIS	5000	none	100%
Proposed	5000	20/20	100%
Proposed	5000	20/20*	98.2%

### Performance evaluation

The computational performance of the proposed approach was tested with respect to the Molecular Hashkeys algorithm [4], which builds on the Compass algorithm [17,30]. This selection was based on the fact that both the proposed approach and Molecular Hashkeys (along with its predecessor Compass) seek to define the surface-based similarity between molecules. The distinctions of our approach from these methods lie in how the modelling of molecular shape and field-effects are accomplished as well as in how the similarity is computed. Furthermore, our selection was also motivated by the fact that Compass along with its derivatives have been extensively applied in pharmaceutical research settings and the published results [4,17,30] as well as our own investigations show it to be amongst the most efficient approaches currently available for determining surface-based molecular similarity.

**Figure 2:**
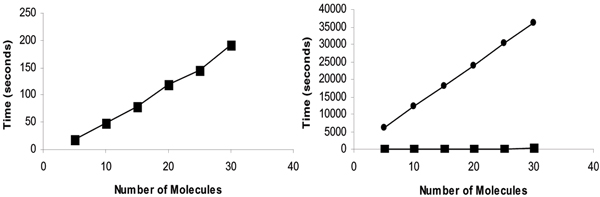
**Comparison of computational performance**. Computational performance of the proposed method (left) and comparison with the Molecular Hashkeys method [13], which is based on the Compass algorithm (right).

In our experiment, 30 molecules from the MDDR collection were compared against each other, with 20 conformers for the model and one for the query. Both the systems reported a 100% recognition rate on this subset of molecules. However, the time requirements were significantly different. A graph plotting the time required for the similarity computation with the proposed technique is shown in Figure 2 (left plot with the data points shown as squares). Figure 2 (right plot) shows a comparison of the performance with the Molecular Hashkeys method (data points corresponding to the Molecular Hashkeys algorithm are shown as circles). On an average, with the proposed technique 120 conformers were processed (histogram generation and matching) every second, while with Molecular Hashkeys, one conformer was matched every two seconds. Both results were obtained on an SGI Indigo2 machine. Another recent commercially available method [7] reports matching speed of 2 minutes per molecule (on a SUN Ultra-30). It should be noted that descriptor generation (estimating the molecular shape and computing the donor/acceptor fields strengths) in both the methods took similar time, averaging around 5 seconds per conformer and was done offline. However, in the current implementation of our approach, histogram generation is done online. Therefore, further speedups are possible by making histogram generation part of the one-time off-line computation.

For a given molecular property and its corresponding property-histogram having *n *bins, computing the similarity of a pair of conformers using the proposed technique (see the Methods section) involves determining the histogram intersection scores for matching the spatial distribution of points corresponding to the each of the aforementioned *n *bins. For each bin, this score is then used to weight the intersection score of the property-histogram. For an encapsulating sphere of circumference *C*, characterizing the spatial distribution requires *O*(*C*) bins. Since histogram intersection is linear in the number of bins, the matching complexity is therefore *O(Cn)*.

Given the size of molecular repositories, a key technical problem is the design of indexing techniques. This is due to the fact that even highly efficient matching techniques, such as the one presented, require distance comparisons which grow linearly with the number of molecules in the database. Indexing techniques can be broadly classified as (1) *spatial access methods*, such as Quadtree [31], R-Tree [32], and KD-Tree [33], which are applicable when items in the repository are represented by a finite set of attributes or features and Euclidean distance between a pair of features/attributes can be defined. Such methods function by using the Euclidean structure of the embedding space to divide repository entities into clusters and avoiding the search of some clusters during retrieval. (2) *distance-based indexing methods*, such as GNAT-Tree [34] and VP-Tree [35], which rely only on pair-wise distances for data retrieval and employ the triangle-inequality for pruning the search space. While a detailed analysis of indexing techniques is beyond the scope of this paper, it is important to note that the performance of spatial access methods is very good for small number of attributes and rapidly degrades as the number of features increase. On the other hand, the efficiency of distance-based indexing is not good for large data collections. This underlines the necessity for developing indexing techniques, such as [36], which utilize specificities of structural data in the design of the indexing strategy.

### Validation through application in structure-activity models

A structure-property model captures the relationship between the bio-chemical properties of a molecule and its physicochemical description [37] by envisaging the biochemical property Φ of a molecule *M*_*i *_as the function of its "chemical constitution":

Φ = *f*(*M*_*i*_)

The basic elements needed for the development of a structure-property model are: (1) Assay results describing the bio-chemical property of interest, (2) a set of parameters describing the molecular structure and its physicochemical attributes, and (3) the learning formulation along with a statistical or machine learning technique.

As part of the validation experiments, similarity information derived using the proposed technique was used to model absorption through an in-vitro cell line. The data set consisted of 30 compounds that were tested using the Caco-2 assay. The Caco-2 (human colon adenocarcinoma cell line) provides a close approximation of *in vivo *absorption and can be used to model the epithelial cell layer barrier and absorption from the intestinal lumen to the blood stream. The assay protocol used in this experiment was designed to measure uni-directional flux and all compounds were analysed at identical initial concentrations. The range of measured values was between 0.0% (no permeation) to 2.8% (maximum permeation) flux units. The set of parameters describing the molecular structure consisted of a 31-dimensional descriptor vector. The first element of this vector was the computed octanol-water partition coefficient (clogP). The remaining thirty elements of the vector were obtained by computing the similarity (using the proposed method) of the molecules tested in the assay to a predefined set of thirty molecules that represented a maximally diverse set of the MDDR collection. The central idea behind such form of molecular description relates to the concept of vector quantization [38] and implicit dimensionality reduction [4]. A backpropagation-based neural network with one hidden layer was then used to estimate the unknown continuous relationship between the molecules and Caco-2 permeation.

Two measures were used for evaluation of the results. The first is a ratio-scale measure called cross-validated *r*^2 ^and shows how well the model predicts data that was *not used *during model construction. This measure is defined as (Eq. (2)):

r2=1−∑i(Vi−Pi)2∑i(Vi−V¯)2
 MathType@MTEF@5@5@+=feaafiart1ev1aaatCvAUfKttLearuWrP9MDH5MBPbIqV92AaeXatLxBI9gBaebbnrfifHhDYfgasaacH8akY=wiFfYdH8Gipec8Eeeu0xXdbba9frFj0=OqFfea0dXdd9vqai=hGuQ8kuc9pgc9s8qqaq=dirpe0xb9q8qiLsFr0=vr0=vr0dc8meaabaqaciaacaGaaeqabaqabeGadaaakeaacqWGYbGCdaahaaWcbeqaaiabikdaYaaakiabg2da9iabigdaXiabgkHiTmaalaaabaWaaabuaeaacqGGOaakcqWGwbGvdaWgaaWcbaGaemyAaKgabeaakiabgkHiTiabdcfaqnaaBaaaleaacqWGPbqAaeqaaOGaeiykaKYaaWbaaSqabeaacqaIYaGmaaaabaGaemyAaKgabeqdcqGHris5aaGcbaWaaabuaeaacqGGOaakcqWGwbGvdaWgaaWcbaGaemyAaKgabeaakiabgkHiTiqbdAfawzaaraGaeiykaKYaaWbaaSqabeaacqaIYaGmaaaabaGaemyAaKgabeqdcqGHris5aaaaaaa@4A34@

Here, *V*_*i *_is the experimentally determined property of the molecule *M*_*i*_, *P*_*i *_is its predicted property, and V¯
 MathType@MTEF@5@5@+=feaafiart1ev1aaatCvAUfKttLearuWrP9MDH5MBPbIqV92AaeXatLxBI9gBaebbnrfifHhDYfgasaacH8akY=wiFfYdH8Gipec8Eeeu0xXdbba9frFj0=OqFfea0dXdd9vqai=hGuQ8kuc9pgc9s8qqaq=dirpe0xb9q8qiLsFr0=vr0=vr0dc8meaabaqaciaacaGaaeqabaqabeGadaaakeaacuWGwbGvgaqeaaaa@2DF9@ is the mean experimental property value. The second measure is an ordinal measure called Kendall's *τ*, which shows how well the *ordering *of the data is preserved during prediction by the model. This measure, computed for *n *molecules, is defined as:

τ=|correctly orderd pairs|−|incorrectly ordered pairs|n(n−1)/2
 MathType@MTEF@5@5@+=feaafiart1ev1aaatCvAUfKttLearuWrP9MDH5MBPbIqV92AaeXatLxBI9gBaebbnrfifHhDYfgasaacH8akY=wiFfYdH8Gipec8Eeeu0xXdbba9frFj0=OqFfea0dXdd9vqai=hGuQ8kuc9pgc9s8qqaq=dirpe0xb9q8qiLsFr0=vr0=vr0dc8meaabaqaciaacaGaaeqabaqabeGadaaakeaaiiGacqWFepaDcqGH9aqpdaWcaaqaaiabcYha8jabbogaJjabb+gaVjabbkhaYjabbkhaYjabbwgaLjabbogaJjabbsha0jabbYgaSjabbMha5jabbccaGiabb+gaVjabbkhaYjabbsgaKjabbwgaLjabbkhaYjabbsgaKjabbccaGiabbchaWjabbggaHjabbMgaPjabbkhaYjabbohaZjabcYha8jabgkHiTiabcYha8jabbMgaPjabb6gaUjabbogaJjabb+gaVjabbkhaYjabbkhaYjabbwgaLjabbogaJjabbsha0jabbYgaSjabbMha5jabbccaGiabb+gaVjabbkhaYjabbsgaKjabbwgaLjabbkhaYjabbwgaLjabbsgaKjabbccaGiabbchaWjabbggaHjabbMgaPjabbkhaYjabbohaZjabcYha8bqaaiabd6gaUjabcIcaOiabd6gaUjabgkHiTiabigdaXiabcMcaPiabc+caViabikdaYaaaaaa@7CC5@

Kendall's *τ *is determined by considering all pairs of predicted absorption values and the corresponding actual absorption values (as determined experimentally). A pair of predicted values is deemed to be correctly ordered if the ordering coincides with that of the experimentally derived values. The numerator in Eq. 3 is the difference between the numbers of correctly and incorrectly ordered pairs. The denominator denotes the number of all possible pairs. Thus, if all pairs are correctly ordered, the maximum value of *τ *= 1 is obtained. On the other hand, the minimum value of *τ *= -1 is obtained if none of the pairs of predicted values retain the experimentally derived ordering. An ordinal measure, such as Kendall's *τ*, reflects how well the model can predict the ordering (or prioritisation) of the molecules. This provides an alternate way to assess the model as compared to measuring the numeric predictive accuracy. Therefore, using a combination of the above measures allows a multifaceted approach to model evaluation.

The assay values for twenty of the thirty compounds were made available for model construction and constituted the learning phase for the neural network. As part of the model construction step, the complete cross-correlation matrix of the descriptors was computed and the top eight least correlated descriptors used to learn the (empirical) mapping between the molecules and their permeability values. Learning was stopped when the cross-validated error became lower than a predefined threshold.

**Figure 3:**
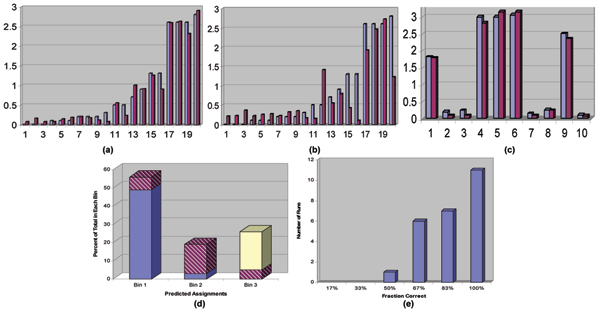
**Performance in structure-property modelling**. Performance, comparison, and analysis of the proposed method in structure-property modelling. In (a) – (c), permeation of each compound is depicted by two adjacent bars with predicted values represented by light-blue bars on the left and measured values represented by the dark maroon bars on the right. The numbers on the X-axis identify each molecule used in the experiment and the Y-axis corresponds to the permeation values, measured in terms of flux-units. (a) Prediction results on the training set in a leave-one-out setting with the proposed method, (b) Prediction results on the training set in a leave-one-out setting with the similarity algorithm [13], (c) Performance of the proposed method on the test set. Figures 3(d) and 3(e) present leave-n-out cross-validated results demonstrating the robustness of the predictive model obtained using the proposed method. The correctness of the assignment of the molecules to the three classes "low permeability", "medium permeability", and "high permeability" is shown in (d), while the distribution of the prediction results in shown in (e).

We begin by presenting the analysis of the method's performance in a leave-one-out cross-validated setting on the training set. In this setting, one compound was randomly excluded from the training set and the remaining compounds used to learn a model that predicted the permeability for the excluded compound. The results are shown in Figures 3(a) and 3(b). The numbers on the X-axis identify each of the molecules in the test set and the Y-axis shows the permeation values in terms of flux-units. Figure 3(a) shows the predictive performance of the model constructed with the proposed similarity measure. In this case the cross-validated *r*^2 ^equalled 0.97 and the value for Kendall's *τ *was 0.65. In Figure 3(b), results are shown for the identical problem setting, where the only exception was the use of the Molecular Hashkeys algorithm for computing the similarity of the molecules. For the best model learnt based on descriptors generated using Molecular Hashkeys, the value for cross-validated *r*^2 ^equalled 0.64 and Kendall's *τ *equalled 0.29. It should be emphasized that in both experiments an identical learning algorithm (single hidden layer neural network with back-propagation) was used and the only distinction was in the similarity values (due to the different algorithms used for determining them). We also note that the relatively low value for Kendall's *τ *(as compared to the cross-validated *r*^2^) occurred because the original data had compounds showing no absorption. The models that were derived typically assigned very low (albeit non-zero) absorption values to these molecules, thus leading to lower values for Kendall's *τ *. The model based on similarity values derived using Molecular Hashkeys also exhibited ordering inconsistencies across the entire range of absorption values. Finally, Figure 3(c) shows the performance of the structure-activity model obtained using the proposed method, on the test set of 10 molecules. Here, the X-axis identifies each of the ten molecules in the test set, while the Y-axis corresponds to the permeation values.

In Figure 3(d) – (e), we present an analysis of the method's performance in a leave-n-out cross-validated setting. The goal of this experiment was to examine the robustness of the model under conditions where a significant number of samples get left out during the model construction stage. During each iteration of the experiment, 7 of the 20 molecules were randomly excluded. The remaining 13 molecules were then used for model construction and for predicting the absorption values of the 7 excluded molecules. The results are based on the performance of the model in 25 iterations of the leave-n-out experiment. The number of iterations is arbitrarily selected. To help visualize the results, the absorption values and predictions are grouped into three bins: Bin 1 corresponds to molecules exhibiting poor absorption (defined to be less than 0.5% flux units), Bin 2 corresponds to molecules that exhibited medium absorption (between 0.5% and 1.0% flux), and Bin 3 corresponds to molecules that showed high permeation (greater than 1.0% flux). The bar-chart in Figure 3(d) shows the number of incorrect bin assignments that were made: Over the 25 iterations, 85% of the overall bin assignments were correct and in 15% of the assignments, an error of one adjacent bin was observed (i.e. a compound with low absorption got assigned to the medium absorption bin or vice-versa, or a medium absorption compound was assigned to the high absorption bin). However, in none of the iterations, was a poorly absorbed compound predicted to be a highly absorbed one or a highly absorbed compound predicted to be a poorly absorbed one. Figure 3(e), presents the distribution of the prediction results across the 25 iterations of the leave-n-out cross-validation experiment: 11 of the 25 iterations resulted in perfect bin assignments and 7 of the 25 iterations had 83% correct bin assignments. Further, 6 of the iterations had 67% accurate assignments and only one of the 25 iterations had 50% accuracy in bin assignments. These statistics indicate the high consistency in the prediction performance of the model across variations in the training set.

## Conclusion

In this paper, we considered the problem of defining similarity between molecules based on complex surface-based representations. Such representations capture the physics of the molecules better than commonly used molecular-graph-based approaches and can therefore have significant relevance in molecular query-retrieval, similarity-based exploration of structural space, and structure-activity modelling. We have presented a novel approach for defining a standard coordinate system for describing complex surface-based molecular descriptions. For computing the similarity of molecules, we propose a novel formulation of histogram intersection which can take into account the distribution of surface properties in 3D space. Experimental results indicate that the similarity formulation can be used for highly-accurate query-retrieval and outperforms, in terms of computational speed, both existing research and commercially available solutions. The proposed approach was also validated by applying it in building structure-activity models for complex bio-chemical properties. The efficacy and computational efficiency of the proposed approach underline the important role it can play in querying and exploration of large molecular repositories.

## Methods

We begin this section by describing how the molecular surfaces are derived and how at each point of the surface, donor and acceptor fields are defined. Next, the concept of a standard coordinate system for describing molecular surfaces is introduced. In this subsection we discuss the Gauss map and its derivatives: the Extended Gaussian Image and the Spherical Attribute Image. We subsequently describe how a sphere encapsulating the molecule is deformed to map the molecular surface to a standard spherical coordinate system. In the final sub-section, the histogram-intersection based surface matching algorithm is described and illustrated using a simple example.

### Computing the molecular surface and surface properties

Starting from the atomic coordinates, the molecular surface (Connolly surface) is obtained by using the program MSRoll [39]. The geometric information provided by the molecular surface is complemented by calculating the donor field and acceptor field (due to H-bond donor and H-bond acceptor atoms) of the molecule at each surface point. The choice of these descriptors is due to their importance in various molecular interactions and their correlation with other surface-based properties such as polar surface area [40].

The measurement of the donor field is done using the following three step procedure:

#### Step 1

The Hydrogen-bond donor atoms in the molecule are identified. Typically these are Nitrogen or Oxygen atoms with hydrogen on them. Other ways of identification like the PATTY-rule [13] can also be used in this stage.

#### Step 2

The donor field is defined as an isotropic Gaussian distribution and the field at point P_j _due to an atom at position X_i _having van der Walls radii *r*_*i *_is defined as [7]:

f(Pj,Xi)=(a22πri2)32exp⁡(−a22ri2|Xi−Pj|2)
 MathType@MTEF@5@5@+=feaafiart1ev1aaatCvAUfKttLearuWrP9MDH5MBPbIqV92AaeXatLxBI9gBaebbnrfifHhDYfgasaacH8akY=wiFfYdH8Gipec8Eeeu0xXdbba9frFj0=OqFfea0dXdd9vqai=hGuQ8kuc9pgc9s8qqaq=dirpe0xb9q8qiLsFr0=vr0=vr0dc8meaabaqaciaacaGaaeqabaqabeGadaaakeaacqWGMbGzcqGGOaakcqWGqbaudaWgaaWcbaGaemOAaOgabeaakiabcYcaSiabdIfaynaaBaaaleaacqWGPbqAaeqaaOGaeiykaKIaeyypa0ZaaeWaaeaadaWcaaqaaiabdggaHnaaCaaaleqabaGaeGOmaidaaaGcbaGaeGOmaidcciGae8hWdaNaemOCai3aa0baaSqaaiabdMgaPbqaaiabikdaYaaaaaaakiaawIcacaGLPaaadaahaaWcbeqaamaalaaabaGaeG4mamdabaGaeGOmaidaaaaakiGbcwgaLjabcIha4jabcchaWnaabmaabaWaaSaaaeaacqGHsislcqWGHbqydaahaaWcbeqaaiabikdaYaaaaOqaaiabikdaYiabdkhaYnaaDaaaleaacqWGPbqAaeaacqaIYaGmaaaaaOGaeiiFaWNaemiwaG1aaSbaaSqaaiabdMgaPbqabaGccqGHsislcqWGqbaudaWgaaWcbaGaemOAaOgabeaakiabcYha8naaCaaaleqabaGaeGOmaidaaaGccaGLOaGaayzkaaaaaa@5CAD@

In Eq. (4) *a *is a scale factor for the radii. The value of a = 2, for which 90% of the electron density lies inside the van-der walls radius of the atom, is used in all the experiments.

#### Step 3

At a given surface point P_*j*_, first, the field strength for each donor atom is computed. The direction of each field is given by a unit vector obtained by joining the corresponding atom to P_*j*_. The resultant donor field at P_*j *_is subsequently defined as the vector sum of all donor field vectors at this point.

The acceptor field is analogously determined. Typically Nitrogen or Oxygen atoms with a lone pair of electrons are considered as acceptors.

### A standard coordinate system for surface-based molecular representations

A pre-requisite for comparing molecules described using surface-based representations is the capability to map points on the curved molecular surface to points on a standard coordinate system. Such a mapping was derived by Gauss [41], by using surface orientations to map points on an arbitrary curved surface to a standard coordinate system defined on a unit sphere. This mapping is formally referred to as the Gauss map and can be defined as follows:

#### Definition 1

**Figure 4:**
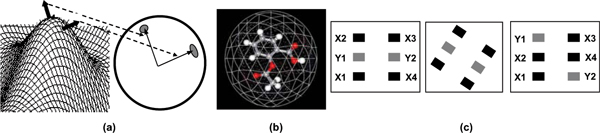
**Illustration of the principle concepts in the proposed molecular representation and matching**. (a) The Gauss Map, (b) Embedding of a molecule in the tessellated sphere, (c) Intuition behind the surface matching approach: The three distributions contain an identical number of black and grey squares and can not be disambiguated by a property (colour)-based histogram. However, a histogram of pair-wise distances between similar colored squares, which captures their spatial distribution, can distinguish the third distribution from the first two. Such a characterization has the added advantage of being invariant to Euclidean transformations of the distribution.

Let *G *⊂ *R*^3 ^be an oriented surface in Euclidean space. Further, let *S *be a unit sphere, called the Gaussian sphere. The Gauss map *M *is the mapping *M *: *G *→ *S*, where the surface normal for each point on the surface *G *is translated to the origin of the sphere *S *and the end points of each normal lie on the surface of the Gaussian sphere *S *(see Figure 4(a) for an illustration).

The *Extended Gaussian Image *(EGI), is a derivative of the Gauss Map and is obtained from it by assuming that the surface *G *is evenly sampled into patches and that each surface normal is associated with a single unit of mass which it votes to the corresponding point on the Gaussian sphere. The distribution of the mass on the surface of the Gaussian sphere, obtained in this fashion, depends on the shape of the underlying surface and constitutes the EGI. The EGI possesses certain important characteristics that include: (i) If two convex objects have the same EGI, they are provably congruent (the Minkowski theorem [42]), (ii) As an object rotates, its EGI rotates in the same manner, (iii) The EGI mass on the Gaussian sphere is inverse of the Gaussian curvature of the underlying object surface, and (iv) The centre of mass of the EGI lies at the origin of the Gaussian sphere.

The properties of the EGI, especially the Minkowski theorem provide the foundations for representing and comparing surface-based description of objects. However, an inherent problem of EGI-type mappings is their dependence on the Gauss map which is non-unique for non-convex shapes. Because of this, more than two points on an object surface may be mapped on the same point on the Gaussian sphere. Unfortunately, many molecules in their stable conformations induce surfaces that are non-convex and therefore the direct application of techniques from the EGI family is precluded for their representation and matching. To address this problem, we utilize the idea of the *Spherical Attribute Image *(SAI) [43], where a geodesic surface is iteratively defined to fit the underlying surface. We begin by placing the molecule inside a semi-regularly tessellated sphere (Figure 4(c)), which is obtained by subdivisions of the triangular sides of a 20-side icosahedron into sub-triangles. The placement of the molecule is done such that its centre of mass coincides with the centre of the sphere. The spherical surface is then modelled as a mechanical system and iteratively deformed to fit the molecular surface. The deformations are subject to a local regularity constraint [44] that ensures uniformity in measurement of the molecular surface and invariance to rotation of the molecule. The convergence of the deformations yield a fit of the deformable surface to the molecular surface and thus provide a one-to-one mapping between points on the molecular surface and points on the encapsulating sphere. The distance of a point on the sphere to its corresponding position on the molecular surface is then used to estimate the surface shape. Further, the donor and acceptor field values at a specific point on the molecular surface are mapped to its corresponding point on the sphere. At the conclusion of this step, each point on the sphere contains three values, characterizing respectively (1) the shape of the underlying surface, (2) the donor field strength, and (3) the acceptor field strength, of the underlying surface point.

### Comparing surface-based molecular representations

We seek to define the similarity of two molecules in terms of the similarity of their surface-property distributions, described using histograms. The technique of *histogram intersection *[45] can be used to rapidly compare the empirical similarity of these distributions. Given the data points corresponding to two distributions, the basic idea consists of quantizing the range of values in fixed size bins. Subsequently, the common number of data points across all the bins is determined and normalized by the size of the distribution. Histogram intersection is computationally efficient since its complexity is linear in the number of bins. Furthermore, the method is robust to noise and invariant to translation and rotation of the distributions being compared.

In the case of molecules, it is critical not just to account for the similarity of property distributions, but also the similarity of the spatial distribution of these properties on the molecular surface. Hence, a direct application of histogram intersection to compare the property distributions is by itself, insufficient. This issue is illustrated in Figure 4(c) where three distributions of four black and two grey squares are shown. Our goal is to devise a technique that can distinguish the first and second distributions (which are identical and related by a planar rotation), from the third. Clearly using the histogram of greyscale value of the squares is insufficient, since all the three distributions produce identical greyscale histograms. Intuitively, we would like two distributions to be considered similar when they have *both *similar distribution of values and are similarly distributed spatially.

Our approach uses the distribution of the pair-wise distances between points having similar property values to characterize the spatial distribution of the corresponding molecular property. Furthermore, we use histogram intersection to compute the similarity of the property distributions as well as the similarity of the spatial distributions. In addition to efficient computability and invariance to translations and rotations of the molecule, a significant advantage of this approach is its ability to characterize (and compare) the relative spatial distribution of surface properties, which act as pharmacophores. The main steps of the method are:

#### Step 1

For each specific property of the molecule, such as shape, donor field, or acceptor field, the property values across all the points on the surface of the tessellated sphere are determined. The range of values is then uniformly divided into a predefined number *N *of bins (we use *N *= 100 in all our experiments). Next, the frequency of points lying in each bin is computed. This defines the histogram of the corresponding property distribution. We term such histograms as property-histograms. In general, let P_1_...P_*K *_denote the *K *properties being used to characterize a molecule. In the following, we shall denote by H_*L*_, the property-histogram corresponding to the property *P*_*L*_, *L *∈ [1,..., *K*]. For each property-bin *m *of H_*L*_steps 2–4 are repeated.

#### Step 2

The points contained in property-bin *m *are clustered in terms of their adjacency on the surface of the encapsulating sphere and the centroid of each cluster is determined.

#### Step 3

The geodesic distance between all pairs of centroids is computed. We note that Steps 2–3 constitute a computationally cheaper alternative to computing the distances between all pairs of points in property-bin *m*.

#### Step 4

These distances are quantized in distance-bins which are defined in increments of one Angstrom in the range [0, *C*/2], where *C *denotes the circumference of the encapsulating sphere (measured in Angstroms). Next, the frequency in each distance-bin is computed to come up with the distance-histogram. Thus, there is a distance-histogram corresponding to every bin *m *of a property-histogram H_*L*_. The content of a distance-bin denotes the number of points on the surface of the sphere that lie within a specific distance (equal to the range of the distance-bin) of each other and have values for the property P_*L *_that fall within the range of property-bin *m *in H_*L*_.

#### Step 5

Consider two molecules *M*_1 _and *M*_2_, a property P_*L *_along with the corresponding property histograms HL1
 MathType@MTEF@5@5@+=feaafiart1ev1aaatCvAUfKttLearuWrP9MDH5MBPbIqV92AaeXatLxBI9gBaebbnrfifHhDYfgasaacH8akY=wiFfYdH8Gipec8Eeeu0xXdbba9frFj0=OqFfea0dXdd9vqai=hGuQ8kuc9pgc9s8qqaq=dirpe0xb9q8qiLsFr0=vr0=vr0dc8meaabaqaciaacaGaaeqabaqabeGadaaakeaacqWGibasdaqhaaWcbaGaemitaWeabaGaeGymaedaaaaa@3003@ and HL2
 MathType@MTEF@5@5@+=feaafiart1ev1aaatCvAUfKttLearuWrP9MDH5MBPbIqV92AaeXatLxBI9gBaebbnrfifHhDYfgasaacH8akY=wiFfYdH8Gipec8Eeeu0xXdbba9frFj0=OqFfea0dXdd9vqai=hGuQ8kuc9pgc9s8qqaq=dirpe0xb9q8qiLsFr0=vr0=vr0dc8meaabaqaciaacaGaaeqabaqabeGadaaakeaacqWGibasdaqhaaWcbaGaemitaWeabaGaeGOmaidaaaaa@3005@, and a bin *m *of the property-histograms. Let Dm1L
 MathType@MTEF@5@5@+=feaafiart1ev1aaatCvAUfKttLearuWrP9MDH5MBPbIqV92AaeXatLxBI9gBaebbnrfifHhDYfgasaacH8akY=wiFfYdH8Gipec8Eeeu0xXdbba9frFj0=OqFfea0dXdd9vqai=hGuQ8kuc9pgc9s8qqaq=dirpe0xb9q8qiLsFr0=vr0=vr0dc8meaabaqaciaacaGaaeqabaqabeGadaaakeaacqWGebardaqhaaWcbaGaemyBa0gabaGaeGymaeZaaSbaaWqaaiabdYeambqabaaaaaaa@318B@ and Dm2L
 MathType@MTEF@5@5@+=feaafiart1ev1aaatCvAUfKttLearuWrP9MDH5MBPbIqV92AaeXatLxBI9gBaebbnrfifHhDYfgasaacH8akY=wiFfYdH8Gipec8Eeeu0xXdbba9frFj0=OqFfea0dXdd9vqai=hGuQ8kuc9pgc9s8qqaq=dirpe0xb9q8qiLsFr0=vr0=vr0dc8meaabaqaciaacaGaaeqabaqabeGadaaakeaacqWGebardaqhaaWcbaGaemyBa0gabaGaeGOmaiZaaSbaaWqaaiabdYeambqabaaaaaaa@318D@ denote the distance-histograms of property-bin *m *for molecules *M*_1 _and *M*_2 _respectively. The similarity *γ*_*m*_, of the spatial distribution of points lying in property-bin *m*, for *M*_1 _and *M*_2 _is defined as the histogram intersection of Dm1L
 MathType@MTEF@5@5@+=feaafiart1ev1aaatCvAUfKttLearuWrP9MDH5MBPbIqV92AaeXatLxBI9gBaebbnrfifHhDYfgasaacH8akY=wiFfYdH8Gipec8Eeeu0xXdbba9frFj0=OqFfea0dXdd9vqai=hGuQ8kuc9pgc9s8qqaq=dirpe0xb9q8qiLsFr0=vr0=vr0dc8meaabaqaciaacaGaaeqabaqabeGadaaakeaacqWGebardaqhaaWcbaGaemyBa0gabaGaeGymaeZaaSbaaWqaaiabdYeambqabaaaaaaa@318B@ and Dm2L
 MathType@MTEF@5@5@+=feaafiart1ev1aaatCvAUfKttLearuWrP9MDH5MBPbIqV92AaeXatLxBI9gBaebbnrfifHhDYfgasaacH8akY=wiFfYdH8Gipec8Eeeu0xXdbba9frFj0=OqFfea0dXdd9vqai=hGuQ8kuc9pgc9s8qqaq=dirpe0xb9q8qiLsFr0=vr0=vr0dc8meaabaqaciaacaGaaeqabaqabeGadaaakeaacqWGebardaqhaaWcbaGaemyBa0gabaGaeGOmaiZaaSbaaWqaaiabdYeambqabaaaaaaa@318D@:

γm=(∩(Dm1L,Dm2L)+∩(Dm2L,Dm1L))/2
 MathType@MTEF@5@5@+=feaafiart1ev1aaatCvAUfKttLearuWrP9MDH5MBPbIqV92AaeXatLxBI9gBaebbnrfifHhDYfgasaacH8akY=wiFfYdH8Gipec8Eeeu0xXdbba9frFj0=OqFfea0dXdd9vqai=hGuQ8kuc9pgc9s8qqaq=dirpe0xb9q8qiLsFr0=vr0=vr0dc8meaabaqaciaacaGaaeqabaqabeGadaaakeaaiiGacqWFZoWzdaWgaaWcbaGaemyBa0gabeaakiabg2da9iabcIcaOiablMIijjabcIcaOiabdseaenaaDaaaleaacqWGTbqBaeaacqaIXaqmdaWgaaadbaGaemitaWeabeaaaaGccqGGSaalcqWGebardaqhaaWcbaGaemyBa0gabaGaeGOmaiZaaSbaaWqaaiabdYeambqabaaaaOGaeiykaKIaey4kaSIaeSykIKKaeiikaGIaemiraq0aa0baaSqaaiabd2gaTbqaaiabikdaYmaaBaaameaacqWGmbataeqaaaaakiabcYcaSiabdseaenaaDaaaleaacqWGTbqBaeaacqaIXaqmdaWgaaadbaGaemitaWeabeaaaaGccqGGPaqkcqGGPaqkcqGGVaWlcqaIYaGmaaa@507B@

In Eq. (5), the average of the two histogram intersections is taken to ensure symmetry. Further (denoting the indexing of the distance-bins by *j*):

∩(Dm1L,Dm2L)=∑j=1C/2min⁡(Dmj1L,Dmj2L)∑j=1C/2Dmj2L   and   ∩(Dm2L,Dm1L)=∑j=1C/2min⁡(Dmj1L,Dmj2L)∑j=1C/2Dmj1L
 MathType@MTEF@5@5@+=feaafiart1ev1aaatCvAUfKttLearuWrP9MDH5MBPbIqV92AaeXatLxBI9gBaebbnrfifHhDYfgasaacH8akY=wiFfYdH8Gipec8Eeeu0xXdbba9frFj0=OqFfea0dXdd9vqai=hGuQ8kuc9pgc9s8qqaq=dirpe0xb9q8qiLsFr0=vr0=vr0dc8meaabaqaciaacaGaaeqabaqabeGadaaakeaacqWIPisscqGGOaakcqWGebardaqhaaWcbaGaemyBa0gabaGaeGymaeZaaSbaaWqaaiabdYeambqabaaaaOGaeiilaWIaemiraq0aa0baaSqaaiabd2gaTbqaaiabikdaYmaaBaaameaacqWGmbataeqaaaaakiabcMcaPiabg2da9maalaaabaWaaabCaeaacyGGTbqBcqGGPbqAcqGGUbGBcqGGOaakcqWGebardaqhaaWcbaGaemyBa02aaSbaaWqaaiabdQgaQbqabaaaleaacqaIXaqmdaWgaaadbaGaemitaWeabeaaaaGccqGGSaalcqWGebardaqhaaWcbaGaemyBa02aaSbaaWqaaiabdQgaQbqabaaaleaacqaIYaGmdaWgaaadbaGaemitaWeabeaaaaGccqGGPaqkaSqaaiabdQgaQjabg2da9iabigdaXaqaaiabdoeadjabc+caViabikdaYaqdcqGHris5aaGcbaWaaabCaeaacqWGebardaqhaaWcbaGaemyBa02aaSbaaWqaaiabdQgaQbqabaaaleaacqaIYaGmdaWgaaadbaGaemitaWeabeaaaaaaleaacqWGQbGAcqGH9aqpcqaIXaqmaeaacqWGdbWqcqGGVaWlcqaIYaGma0GaeyyeIuoaaaGccqqGGaaicqqGGaaicqqGGaaicqqGHbqycqqGUbGBcqqGKbazcqqGGaaicqqGGaaicqqGGaaicqWIPisscqGGOaakcqWGebardaqhaaWcbaGaemyBa0gabaGaeGOmaiZaaSbaaWqaaiabdYeambqabaaaaOGaeiilaWIaemiraq0aa0baaSqaaiabd2gaTbqaaiabigdaXmaaBaaameaacqWGmbataeqaaaaakiabcMcaPiabg2da9maalaaabaWaaabCaeaacyGGTbqBcqGGPbqAcqGGUbGBcqGGOaakcqWGebardaqhaaWcbaGaemyBa02aaSbaaWqaaiabdQgaQbqabaaaleaacqaIXaqmdaWgaaadbaGaemitaWeabeaaaaGccqGGSaalcqWGebardaqhaaWcbaGaemyBa02aaSbaaWqaaiabdQgaQbqabaaaleaacqaIYaGmdaWgaaadbaGaemitaWeabeaaaaGccqGGPaqkaSqaaiabdQgaQjabg2da9iabigdaXaqaaiabdoeadjabc+caViabikdaYaqdcqGHris5aaGcbaWaaabCaeaacqWGebardaqhaaWcbaGaemyBa02aaSbaaWqaaiabdQgaQbqabaaaleaacqaIXaqmdaWgaaadbaGaemitaWeabeaaaaaaleaacqWGQbGAcqGH9aqpcqaIXaqmaeaacqWGdbWqcqGGVaWlcqaIYaGma0GaeyyeIuoaaaaaaa@A8EF@

#### Step 6

The similarity of two molecules *M*_1 _and *M*_2_, in context of the property P_*L *_is denoted by *Sim*_*L*_(*M*_1_, *M*_2_) and is defined as the histogram intersection of the corresponding property-histograms HL1
 MathType@MTEF@5@5@+=feaafiart1ev1aaatCvAUfKttLearuWrP9MDH5MBPbIqV92AaeXatLxBI9gBaebbnrfifHhDYfgasaacH8akY=wiFfYdH8Gipec8Eeeu0xXdbba9frFj0=OqFfea0dXdd9vqai=hGuQ8kuc9pgc9s8qqaq=dirpe0xb9q8qiLsFr0=vr0=vr0dc8meaabaqaciaacaGaaeqabaqabeGadaaakeaacqWGibasdaqhaaWcbaGaemitaWeabaGaeGymaedaaaaa@3003@ and HL2
 MathType@MTEF@5@5@+=feaafiart1ev1aaatCvAUfKttLearuWrP9MDH5MBPbIqV92AaeXatLxBI9gBaebbnrfifHhDYfgasaacH8akY=wiFfYdH8Gipec8Eeeu0xXdbba9frFj0=OqFfea0dXdd9vqai=hGuQ8kuc9pgc9s8qqaq=dirpe0xb9q8qiLsFr0=vr0=vr0dc8meaabaqaciaacaGaaeqabaqabeGadaaakeaacqWGibasdaqhaaWcbaGaemitaWeabaGaeGOmaidaaaaa@3005@, where the intersection score for each property-bin *m *is weighted by *γ*_*m*_. Formally:

SimL(M1,M2)=∩(HL1,HL2).γ+∩(HL2,HL1).γ2
 MathType@MTEF@5@5@+=feaafiart1ev1aaatCvAUfKttLearuWrP9MDH5MBPbIqV92AaeXatLxBI9gBaebbnrfifHhDYfgasaacH8akY=wiFfYdH8Gipec8Eeeu0xXdbba9frFj0=OqFfea0dXdd9vqai=hGuQ8kuc9pgc9s8qqaq=dirpe0xb9q8qiLsFr0=vr0=vr0dc8meaabaqaciaacaGaaeqabaqabeGadaaakeaacqWGtbWucqWGPbqAcqWGTbqBdaWgaaWcbaGaemitaWeabeaakiabcIcaOiabd2eannaaBaaaleaacqaIXaqmaeqaaOGaeiilaWIaemyta00aaSbaaSqaaiabikdaYaqabaGccqGGPaqkcqGH9aqpdaWcaaqaaiablMIijjabcIcaOiabdIeainaaDaaaleaacqWGmbataeaacqaIXaqmaaGccqGGSaalcqWGibasdaqhaaWcbaGaemitaWeabaGaeGOmaidaaOGaeiykaKIaeiOla4ccciGae83SdCMaey4kaSIaeSykIKKaeiikaGIaemisaG0aa0baaSqaaiabdYeambqaaiabikdaYaaakiabcYcaSiabdIeainaaDaaaleaacqWGmbataeaacqaIXaqmaaGccqGGPaqkcqGGUaGlcqWFZoWzaeaacqaIYaGmaaaaaa@560E@

Where (indexing the bins of the property-histogram H_*L *_by the variable *m*), the intersection of the property-histograms of two arbitrary molecules *M*_*a *_and *M*_*b *_is defined as:

∩(HLa,HLb)γ=∑m=1Nmin⁡(HLma,HLmb)×γm∑m=1NHLmb
 MathType@MTEF@5@5@+=feaafiart1ev1aaatCvAUfKttLearuWrP9MDH5MBPbIqV92AaeXatLxBI9gBaebbnrfifHhDYfgasaacH8akY=wiFfYdH8Gipec8Eeeu0xXdbba9frFj0=OqFfea0dXdd9vqai=hGuQ8kuc9pgc9s8qqaq=dirpe0xb9q8qiLsFr0=vr0=vr0dc8meaabaqaciaacaGaaeqabaqabeGadaaakeaacqWIPisscqGGOaakcqWGibasdaqhaaWcbaGaemitaWeabaGaemyyaegaaOGaeiilaWIaemisaG0aa0baaSqaaiabdYeambqaaiabdkgaIbaakiabcMcaPGGaciab=n7aNjabg2da9maalaaabaWaaabCaeaacyGGTbqBcqGGPbqAcqGGUbGBcqGGOaakcqWGibasdaqhaaWcbaGaemitaW0aaSbaaWqaaiabd2gaTbqabaaaleaacqWGHbqyaaGccqGGSaalcqWGibasdaqhaaWcbaGaemitaW0aaSbaaWqaaiabd2gaTbqabaaaleaacqWGIbGyaaGccqGGPaqkcqGHxdaTcqWFZoWzdaWgaaWcbaGaemyBa0gabeaaaeaacqWGTbqBcqGH9aqpcqaIXaqmaeaacqWGobGta0GaeyyeIuoaaOqaamaaqahabaGaemisaG0aa0baaSqaaiabdYeamnaaBaaameaacqWGTbqBaeqaaaWcbaGaemOyaigaaaqaaiabd2gaTjabg2da9iabigdaXaqaaiabd6eaobqdcqGHris5aaaaaaa@6410@

#### Step 7

The similarity between two molecules *M*_1 _and *M*_2 _given *K *properties P_1_...P_*K *_is defined as the average similarity computed over all the *K *properties and is denoted as *Sim*_*full*_(*M*_1_, *M*_2_).

#### Step 8

The overall similarity between the molecules is computed by taking into account molecular conformations; it is defined as the maximum value of *Sim*_*full*_(*M*_1_, *M*_2_) over the set of conformations each of the molecules can attain (see Eq. (9)). The conformations can be generated using a package such as CONCORD [46].

Simoverall(M1,M2)=arg⁡max⁡Ci,Cj {Simfull(C1,C2}
 MathType@MTEF@5@5@+=feaafiart1ev1aaatCvAUfKttLearuWrP9MDH5MBPbIqV92AaeXatLxBI9gBaebbnrfifHhDYfgasaacH8akY=wiFfYdH8Gipec8Eeeu0xXdbba9frFj0=OqFfea0dXdd9vqai=hGuQ8kuc9pgc9s8qqaq=dirpe0xb9q8qiLsFr0=vr0=vr0dc8meaabaqaciaacaGaaeqabaqabeGadaaakeaacqWGtbWucqWGPbqAcqWGTbqBdaWgaaWcbaGaem4Ba8MaemODayNaemyzauMaemOCaiNaemyyaeMaemiBaWMaemiBaWgabeaakiabcIcaOiabd2eannaaBaaaleaacqaIXaqmaeqaaOGaeiilaWIaemyta00aaSbaaSqaaiabikdaYaqabaGccqGGPaqkcqGH9aqpdaWfqaqaaiGbcggaHjabckhaYjabcEgaNjGbc2gaTjabcggaHjabcIha4bWcbaGaem4qam0aaSbaaWqaaiabdMgaPbqabaWccqGGSaalcqWGdbWqdaWgaaadbaGaemOAaOgabeaaaSqabaGccqqGGaaicqGG7bWEcqWGtbWucqWGPbqAcqWGTbqBdaWgaaWcbaGaemOzayMaemyDauNaemiBaWMaemiBaWgabeaakiabcIcaOiabdoeadnaaBaaaleaacqaIXaqmaeqaaOGaeiilaWIaem4qam0aaSbaaSqaaiabikdaYaqabaGccqGG9bqFaaa@64D9@

Where *C*_*i *_and *C*_*j *_denote specific conformers of the molecules *M*_1 _and *M*_2 _respectively. Further, the sets C1={C11,C12,...,C1r} and C2={C21,C22,...,C2r}
 MathType@MTEF@5@5@+=feaafiart1ev1aaatCvAUfKttLearuWrP9MDH5MBPbIqV92AaeXatLxBI9gBaebbnrfifHhDYfgasaacH8akY=wiFfYdH8Gipec8Eeeu0xXdbba9frFj0=OqFfea0dXdd9vqai=hGuQ8kuc9pgc9s8qqaq=dirpe0xb9q8qiLsFr0=vr0=vr0dc8meaabaqaciaacaGaaeqabaqabeGadaaakeaacqWGdbWqdaWgaaWcbaGaeGymaedabeaakiabg2da9iabcUha7jabdoeadnaaDaaaleaacqaIXaqmaeaacqaIXaqmaaGccqGGSaalcqWGdbWqdaqhaaWcbaGaeGymaedabaGaeGOmaidaaOGaeiilaWIaeiOla4IaeiOla4IaeiOla4IaeiilaWIaem4qam0aa0baaSqaaiabigdaXaqaaiabdkhaYbaakiabc2ha9jabbccaGiabdggaHjabd6gaUjabdsgaKjabbccaGiabdoeadnaaBaaaleaacqaIYaGmaeqaaOGaeyypa0Jaei4EaSNaem4qam0aa0baaSqaaiabikdaYaqaaiabigdaXaaakiabcYcaSiabdoeadnaaDaaaleaacqaIYaGmaeaacqaIYaGmaaGccqGGSaalcqGGUaGlcqGGUaGlcqGGUaGlcqGGSaalcqWGdbWqdaqhaaWcbaGaeGOmaidabaGaemOCaihaaOGaeiyFa0haaa@5D33@ respectively denote all the conformations attainable by the molecules *M*_1 _and *M*_2_.

### Illustrative example

We use the point distributions shown in Figure 4(c) to illustrate, in a highly simplified setting, the working of the method. To facilitate the example, we assume that the coordinates of the squares in the left distribution are: X1(0, 0); X2(0, 2); X3(1, 2); X4(1, 0); Y1(0, 1); and Y2(1, 1). Similarly, the coordinates of the squares in the right distribution are: X1(0, 0); X2(0, 1); X3(1, 2); X4(1, 1); Y1(0, 2); and Y2(1, 0). We also note that the middle distribution is identical to the left one and related to it by a rotation. Let *L*, the property of interest be the grey-scale values of the squares. We shall assume that all the dark-coloured squares have a greyscale value of 0, while all the light-coloured squares have a greyscale value of 200. For the sake of simplicity, we also assume that the number of bins *N *equals 2. The property histograms for the three distributions computed in Step-1 are: HL1=HL2=HL3=[〈4〉 〈2〉]
 MathType@MTEF@5@5@+=feaafiart1ev1aaatCvAUfKttLearuWrP9MDH5MBPbIqV92AaeXatLxBI9gBaebbnrfifHhDYfgasaacH8akY=wiFfYdH8Gipec8Eeeu0xXdbba9frFj0=OqFfea0dXdd9vqai=hGuQ8kuc9pgc9s8qqaq=dirpe0xb9q8qiLsFr0=vr0=vr0dc8meaabaqaciaacaGaaeqabaqabeGadaaakeaacqWGibasdaqhaaWcbaGaemitaWeabaGaeGymaedaaOGaeyypa0JaemisaG0aa0baaSqaaiabdYeambqaaiabikdaYaaakiabg2da9iabdIeainaaDaaaleaacqWGmbataeaacqaIZaWmaaGccqGH9aqpcqGGBbWwcqGHPms4cqaI0aancqGHQms8cqqGGaaicqGHPms4cqaIYaGmcqGHQms8cqGGDbqxaaa@461C@. For Steps 2–3 which are repeated for each property-bin of each property-histogram, we simplify by computing all the pair-wise distances between the squares. In Step-4, we find that for each of the three distributions, the smallest (largest) pair-wise distances are: 1 (2.23). Constructing bins of unit size across this range, we obtain the following distance-histograms: D11L=D12L=[〈2〉 〈4〉], D21L=D22L= [〈1〉 〈0〉], D13L= [〈5〉 〈1〉], D23L= [〈0〉 〈1〉]
 MathType@MTEF@5@5@+=feaafiart1ev1aaatCvAUfKttLearuWrP9MDH5MBPbIqV92AaeXatLxBI9gBaebbnrfifHhDYfgasaacH8akY=wiFfYdH8Gipec8Eeeu0xXdbba9frFj0=OqFfea0dXdd9vqai=hGuQ8kuc9pgc9s8qqaq=dirpe0xb9q8qiLsFr0=vr0=vr0dc8meaabaqaciaacaGaaeqabaqabeGadaaakeaacqWGebardaqhaaWcbaGaeGymaedabaGaeGymaeZaaSbaaWqaaiabdYeambqabaaaaOGaeyypa0Jaemiraq0aa0baaSqaaiabigdaXaqaaiabikdaYmaaBaaameaacqWGmbataeqaaaaakiabg2da9iabcUfaBjabgMYiHlabikdaYiabgQYiXlabbccaGiabgMYiHlabbsda0iabgQYiXlabb2faDjabbYcaSiabbccaGiabdseaenaaDaaaleaacqaIYaGmaeaacqaIXaqmdaWgaaadbaGaemitaWeabeaaaaGccqGH9aqpcqWGebardaqhaaWcbaGaeGOmaidabaGaeGOmaiZaaSbaaWqaaiabdYeambqabaaaaOGaeyypa0JaeeiiaaIaee4waSLaeyykJeUaeeymaeJaeyOkJeVaeeiiaaIaeyykJeUaeeimaaJaeyOkJeVaeeyxa0LaeeilaWIaeeiiaaIaemiraq0aa0baaSqaaiabigdaXaqaaiabiodaZmaaBaaameaacqWGmbataeqaaaaakiabg2da9iabbccaGiabcUfaBjabgMYiHlabiwda1iabgQYiXlabbccaGiabgMYiHlabbgdaXiabgQYiXlabb2faDjabbYcaSiabbccaGiabdseaenaaDaaaleaacqaIYaGmaeaacqaIZaWmdaWgaaadbaGaemitaWeabeaaaaGccqGH9aqpcqqGGaaicqqGBbWwcqGHPms4cqqGWaamcqGHQms8cqqGGaaicqGHPms4cqqGXaqmcqGHQms8cqqGDbqxaaa@857D@. In Step-5, consequently, the similarity scores *γ*_*m *_of the spatial distribution of points lying in each of the bins of HL1
 MathType@MTEF@5@5@+=feaafiart1ev1aaatCvAUfKttLearuWrP9MDH5MBPbIqV92AaeXatLxBI9gBaebbnrfifHhDYfgasaacH8akY=wiFfYdH8Gipec8Eeeu0xXdbba9frFj0=OqFfea0dXdd9vqai=hGuQ8kuc9pgc9s8qqaq=dirpe0xb9q8qiLsFr0=vr0=vr0dc8meaabaqaciaacaGaaeqabaqabeGadaaakeaacqWGibasdaqhaaWcbaGaemitaWeabaGaeGymaedaaaaa@3003@ and HL3
 MathType@MTEF@5@5@+=feaafiart1ev1aaatCvAUfKttLearuWrP9MDH5MBPbIqV92AaeXatLxBI9gBaebbnrfifHhDYfgasaacH8akY=wiFfYdH8Gipec8Eeeu0xXdbba9frFj0=OqFfea0dXdd9vqai=hGuQ8kuc9pgc9s8qqaq=dirpe0xb9q8qiLsFr0=vr0=vr0dc8meaabaqaciaacaGaaeqabaqabeGadaaakeaacqWGibasdaqhaaWcbaGaemitaWeabaGaeG4mamdaaaaa@3007@ are: *γ*_1 _= 0.5; *γ*_2 _= 0. In Step-6, the similarity of the first and third distributions is therefore: *Sim*_*L*_(*M*_1_, *M*_3_) = (4 × 0.5 + 2 × 0)/6 = 0.33. The reader may trivially verify that *Sim*_*L*_(*M*_1_, *M*_3_) = 1.0.

## Competing interests

The author declares that they have no competing interests.

## References

[B1] Bohm H-J, Schneider G (2000). Virtual Screening for Bio-Active Molecules: Methods and Principles in Medicinal Chemistry.

[B2] Cramer R, Poss MA, Hermsmeier MA, Caulfield TJ, Kowala MC, Vlentine MT (1999). Prospective Identification of Biologically Active Structures by Topomer Shape Similarity Searching. J Med Chem.

[B3] Kinoshita K, Nakamura H (2003). Identification of protein biochemical functions by similarity search using the molecular surface database eF-site. Protein Science.

[B4] Ghuloum A, Sage C, Jain A (1999). Molecular Hashkeys: A Novel Method for Molecular Characterization and its Application for Predicting Important Pharmaceutical Properties of Molecules. J Med Chem.

[B5] Guba W, Cruciani G, Gundertofte K, Jorgensen F (2000). Molecular Field-Derived Descriptors For The Multivariate Modeling of Pharmacokinetic Data. Molecular Modeling and Prediction of Bioactivity.

[B6] Kubinyi H, Folkers G, Martin Y (1998). 3D QSAR in Drug Design.

[B7] Labute P, Williams C (2001). Flexible Alignment of Small Molecules. J Med Chem.

[B8] Norel R, Fischer D, Wolfson H, Nussinov R (1994). Molecular Surface-Recognition by a Computer Vision-Based Technique. Protein Engineering.

[B9] Wiener H (1947). Structural determination of Paraffin Boiling Points. J of Am Chem Soc.

[B10] Randic M (1975). On Characterization of Molecular Branching. J Am Chem Soc.

[B11] Pearlman RS, Smith KM (1999). Metric validation and the receptor-relevant subspace concept. J Chem Inf Comput Sci.

[B12] Silverman BD, Platt DE (1996). Comparative molecular moment analysis (CoMMA): 3D-QSAR without molecular superposition. J Med Chem.

[B13] Bush B, Sheridan R (1993). PATTY: A programmable Atom Typer and Languagefor Automatic Classification of Atoms in a Molecular Database. J Chem Inf Comp Sci.

[B14] Nikolova N, Jaworska J (2003). Approaches to measure chemical similarity – a review. QSAR Comb Sci.

[B15] Carbo-Dorca R, Girones X, Mezey PG (2001). Fundamentals of Molecular Similarity.

[B16] Barnard J, Downs G, Willett P, Bohm H-J, Schneider G (2000). Descriptor-Based Similarity Measures for Screening Chemical Databases. Virtual Screening for Bio-Active Molecules, Methods and Principles in Medicinal Chemistry.

[B17] Jain A, Koile K, Chapman D (1994). Compass: Predicting Biological Activity from Molecular Surface Properties. Performance Comparison on a Steroid Benchmark. J Med Chem.

[B18] Bemis G, Kuntz I (1992). A fast and efficient method for 2D and 3D molecular shape description. J of Comp -Aided Mol Design.

[B19] Deshpande M, Kuramochi M, Wale N, Karypis G (2005). Frequent Sub-Structure-Based Approaches for Classifying Chemical Compounds. IEEE Trans Knowl Data Eng.

[B20] Raymond J, Gardiner E, Willett P (2002). RASCAL: Calculation of Graph Similarity using Maximum Common Edge Subgraphs. The Computer Journal.

[B21] http://www.mdli.com.

[B22] Chen J, Swamidass SJ, Dou Y, Bruamd J, Baldi P (2005). ChemDB: A Public Database of Small Molecules and Related Chemoinformatics Resources. Bioinformatics.

[B23] Holm L, Sander C (1995). Dali: A network tool for protein structure comparison. Trends Biochem Sci.

[B24] Krissinel E, Henrick K (2004). Secondary Structure Matching (SSM), a new tool for fast protein alignment in three dimensions. Acta crystallogr D Biol Crystallogr.

[B25] Orengo CA, Taylor WR (1990). Protein Structure Alignment. J Theorl Biol.

[B26] Gerstein M, Levitt M (1998). Comprehensive assessment of automatic structural alignment against a manual standard, the Scop classification of proteins. Protein Sci.

[B27] Shindyalov IN, Bourne PE (1998). Protein structure alignment by incremental combinatorial extension (CE) of the optimal path. Protein Eng.

[B28] Singh A, Brutlag D (1997). Hierarchical protein structure superposition using both secondary structure and atomic representations. Proc 5th Intr Conf on Intell Syst for Mol Biol (ISMB).

[B29] Kleywegt GJ (1996). Use of non-crystallographic symmetry in protein structure refinement. Acta Crystallogr D Biol Crystallogr.

[B30] Jain A, Dietterich T, Lathrop R, Chapman D, Critchlow R, Bauer B, Webster T, Lozano-Perez T (1994). Compass: A Shape-Based Machine Learning Tool for Drug Design. Journal of Computer Aided Molecular Design.

[B31] Samet H (1984). The Quadtree and Related Hierarchical Data Structures. ACM Computing Surveys.

[B32] Guttman A (1984). R-tree: A Dynamic Index Structure for Spatial Searching. ACM SIGMOD.

[B33] Bently J, Friedman J (1979). Data Structures for Range Searching. ACM Computing Surveys.

[B34] Brin S (1995). Nearest Neighbor Search in Large Metric Spaces. VLDB.

[B35] Yanilos P (1993). Data Structures and Algorithms for Nearest Neighbor Search in General Metric Spaces. SODA.

[B36] Camoglu O, Kahveci T, Singh A (2004). Index-based Similarity Search for Protein Structure Databases. J Bioinform Comput Biol.

[B37] Livingstone DJ (2000). The Characterization of Chemical Structures Using Molecular Properties. A Survey. J Chem Inf Comput Sci.

[B38] Cherkassky VF, Mulier F (1998). Learning From Data.

[B39] Connolly ML (1993). The Molecular Surface Package. J Mol Graph.

[B40] Veber D, Johnson S, Cheng H-Y, Smith B, Ward K, Kopple K (2002). Molecular Properties that Influence the Oral Bioavailability of Drug Candidates. J Med Chem.

[B41] Gauss KF (1965). General Investigation of Curved Surfaces.

[B42] Lysternik LA (1963). Convex Figures and Polyhedra.

[B43] Hebert M, Ikeuchi K, Delingette H (1995). A Spherical Representation for Recognition of Free-Form Surfaces. IEEE Trans on Pattern Analysis and Machine Intelligence.

[B44] Singh R (2004). Reasoning About Molecular Similarity and Properties. Proc IEEE Comput Syst Bioinform Conf.

[B45] Swain M, Ballard D (1991). Color Indexing. Int J of Comp Vision.

[B46] http://www.tripos.com.

